# Thermo-mechanical properties of 3D printed photocurable shape memory resin for clear aligners

**DOI:** 10.1038/s41598-022-09831-4

**Published:** 2022-04-15

**Authors:** Se Yeon Lee, Hoon Kim, Hyun-Joong Kim, Chooryung J. Chung, Yoon Jeong Choi, Su-Jung Kim, Jung-Yul Cha

**Affiliations:** 1 Department of Orthodontics, College of Dentistry, Yonsei University, Seoul, Korea; 2 Research Institute of Agriculture and Life Sciences, College of Agriculture and Life Sciences, Seoul National University, Seoul, Korea; 3 Graphy Inc., Graphy R&D Center, Seoul, Korea; 4 Laboratory of Adhesion and Bio-Composites, Department of Agriculture, Forestry and Bioresources, Seoul National University, Seoul, Korea; 5 Department of Orthodontics, Gangnam Severance Hospital, Institute of Craniofacial Deformity, College of Dentistry, Yonsei University, Seoul, Korea; 6 Department of Orthodontics, Institute of Craniofacial Deformity, College of Dentistry, Yonsei University, 50-1 Yonsei-ro, Seodaemun-gu, Seoul, 03722 Korea; 7 Department of Orthodontics, Kyung Hee University School of Dentistry, Seoul, Korea

**Keywords:** Biomedical materials, Orthodontics, Dental materials

## Abstract

To overcome the limitations of the conventional vacuum thermoforming manufacturing method, direct 3D printing of clear aligners has been developed. The present study investigated the thermo-mechanical and viscoelastic properties of a photocurable resin TC-85, which is a new material for the direct 3D printed clear aligners, comparing to a conventional thermoplastic material polyethylene terephthalate glycol. Dynamic mechanical analysis was performed to analyse the mechanical behaviours of the two materials at 37 °C and 80 °C, respectively. Furthermore, the shape memory property of the two materials was evaluated using a U-shape bending test, and the shape recovery ratio for 60 min at 37 °C was calculated. The results indicate that TC-85 can constantly apply a light force to the teeth when used for the 3D printed clear aligners, owing to its flexibility and viscoelastic properties. In addition, it is expected that the force decay induced by repeated insertion of the clear aligners will be reduced and a constant orthodontic force will be maintained. Furthermore, its geometric stability at high temperatures and the shape memory properties provide advantages for the clinical application.

## Introduction

Clear aligners have been investigated since Kesling first introduced a tooth positioner fabricated with an elastic polymer in 1945, to move the teeth without bands, brackets, or wires^1^, while Nahoum reported a ‘vacuum-formed dental contour appliance’ in 1964^2^. Align Technology Inc. (Santa Clara, CA, USA) has collated several concepts across literature and applied computer-aided design (CAD)/computer-aided manufacturing (CAM) technology to the manufacturing process of clear aligners. As a result, the efficiency of manufacturing clear aligners has increased and expanded their range of applications^3^. Accordingly, several efforts have been made to develop advanced materials that are suitable for the CAD/CAM technology.

Currently, various thermoplastic materials such as polyethylene terephthalate glycol (PETG), polypropylene, polycarbonate, thermoplastic polyurethanes (TPU), and copolyester, are being used to manufacture clear aligners^4–6^. Among these, PETG is widely used because of its excellent impact and tear strengths, barrier properties, chemical resistance, and transparency^7^. In addition, TPU with greater elasticity is used by Align Technology Inc. (Santa Clara, CA, USA) to achieve more predictable orthodontic movements by applying a lighter and more constant force^6^. Furthermore, multi-hybrid materials have been developed to overcome the limitations of a single material by improving the physical properties of maximum tensile load-bearing capacity^8^.

The development of new materials and enhancement of the material properties have significantly improved the performance of the clear aligners. However, the clear aligners are still manufactured by the conventional method of vacuum thermoforming biocompatible thermoplastic transparent materials on a dental model^9^, which is a complex manufacturing process requiring considerable time and effort. In addition, geometric inaccuracies are induced during the thermoforming process^10^. The shrinkage and expansion of the material that occurs during the thermoforming process affects the orthodontic force and fit of the clear aligners to the dentition. In a study evaluating the fit of the clear aligners fabricated with thermoplastic materials, all materials showed various amounts of shrinkage and expansion^11^, which can alter their thickness and physical properties. As a result of vacuum thermoforming, a maximum decrease in the thickness of the thermoplastic material from 92.6 to 57.5% has been reported^12,13^, in addition to the changes in various physical properties such as transparency, water absorption, surface hardness, and elastic modulus^14^. The geometric inaccuracy induced by thermoforming makes it difficult for clinicians to predict the performance of the clear aligners and treatment outcomes. When clear aligners are used, different predictions are made based on the type of tooth movement, which are ineffective in the cases of extrusion, rotation, and tipping movement, yielding a low predictability (30%)^15,16^. Therefore, to improve the predictability of the clear aligners, auxiliaries such as attachment, interarch elastics, interproximal reduction, and altered aligner geometries are recommended in clinical practice^15,16^.

To overcome the limitations of the conventional manufacturing method, direct 3D printing of clear aligners with a biocompatible material has been recently attempted. This method requires less time and effort, and also results in fewer geometric inaccuracies^10^. The accuracy, fit, and clinical feasibility of direct 3D printed clear aligners have been evaluated in several studies^10,17^. However, only few studies evaluate the physical and mechanical properties of the materials used for 3D printing. There is no known material that can be 3D printed while fulfilling biocompatibility, translucency, and appropriate mechanical properties^10,18,19^. Recently, a 3D printable biocompatible material that was approved by the Korea Food and Drug Administration (KFDA) and European Commission (EC) has been developed. The explicit chemical structure of the material could not be ascertained due to patent issues, but the results of the attenuated total reflectance-Fourier-transform infrared spectroscopic analysis indicated that the material is an aliphatic vinyl ester-urethane polymer, possibly cross-linked with methacrylate functionalization^20^. However, the mechanical properties of the newly developed material are yet to be evaluated for clinical applications. The mechanical properties and behaviour of 3D printing materials composed of cross-linked polymers are expected to be different compared with conventional thermoplastic materials composed of non-cross-linked polymers used for thermoforming.

In addition to the physical properties such as static mechanical properties, it is important to understand the viscoelastic properties of creep and stress relaxation to increase the predictability of clear aligner performance and treatment outcomes. The clear aligners are worn for a long time and repeatedly inserted in the oral cavity, which generates force decay. Viscoelastic polymers, which are employed to fabricate the clear aligners, exhibit intermediate properties of viscosity and elasticity^5^. Therefore, the behaviour of the clear aligners considerably varies over time under a constant load^21^. Under repeated and constant loads, the deflection of viscoelastic material increases over time (a phenomenon known as creep), and under constant deflection their force gradually decreases (a phenomenon known as stress relaxation)^6^. In a previous study, the thermoplastic materials showed a stress relaxation of approximately 17.9–62% over 24 h at 37 °C and 100% humidity, while the amount of relaxation varied depending on the type of material and the number of layers^6^.

The purpose of the present study is to evaluate the thermo-mechanical and viscoelastic properties of a newly developed photocurable resin (TC-85), a material for direct 3D printing clear aligners. For comparative evaluation, PETG was selected as a control. To test the null hypothesis that there are no differences in the physical properties of the two materials, a static mechanical test and dynamic mechanical analysis (DMA) were performed to evaluate their viscoelastic and mechanical properties in response to temperature. In addition, the shape memory property of the photocurable resin (TC-85) was investigated.

## Results

### Thickness change

The average thickness of the PETG specimens thermoformed on the standardized model was 0.41 mm, which was only 54.7% of the thickness before thermoforming (0.75 mm). The average thickness of the TC-85 specimens manufactured by 3D printing was 0.56 mm, which was 12% higher than the set thickness of 0.5 mm.

### Static mechanical properties

**Figure 1:**
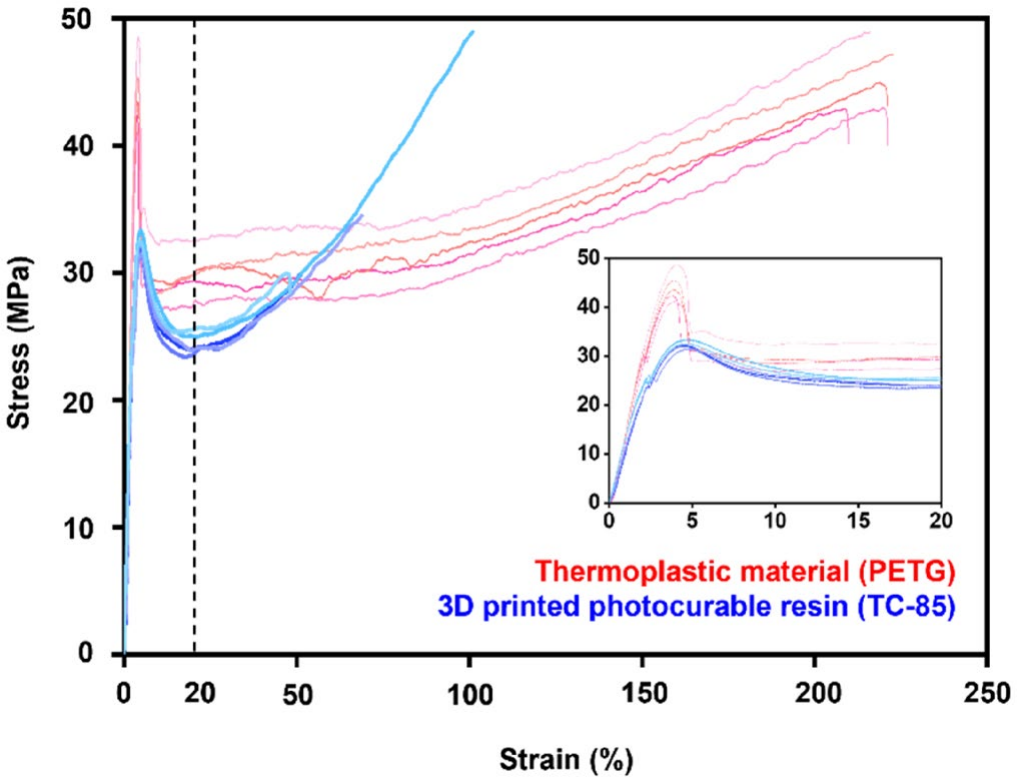
Stress–strain curve of TC-85 and PETG; The small stress–strain curve is an enlargement of the initial range (dash line).

At the yield point, PETG showed 44.20 MPa stress with a strain of 3.92%, while the yield stress of TC-85 was 32.31 MPa with a strain of 4.65% (*p* < 0.01). The elastic moduli of PETG and TC-85 were 1479.54 MPa and 1186.40 MPa, respectively, and the stiffness of PETG was significantly higher (*p* < 0.01). Further, PETG and TC-85 fractured at approximately 232.93% and 62.55% elongation, respectively (Fig. 1).

### Thermo-mechanical cycle properties

#### Stress relaxation and creep at 37 °C

**Figure 2:**
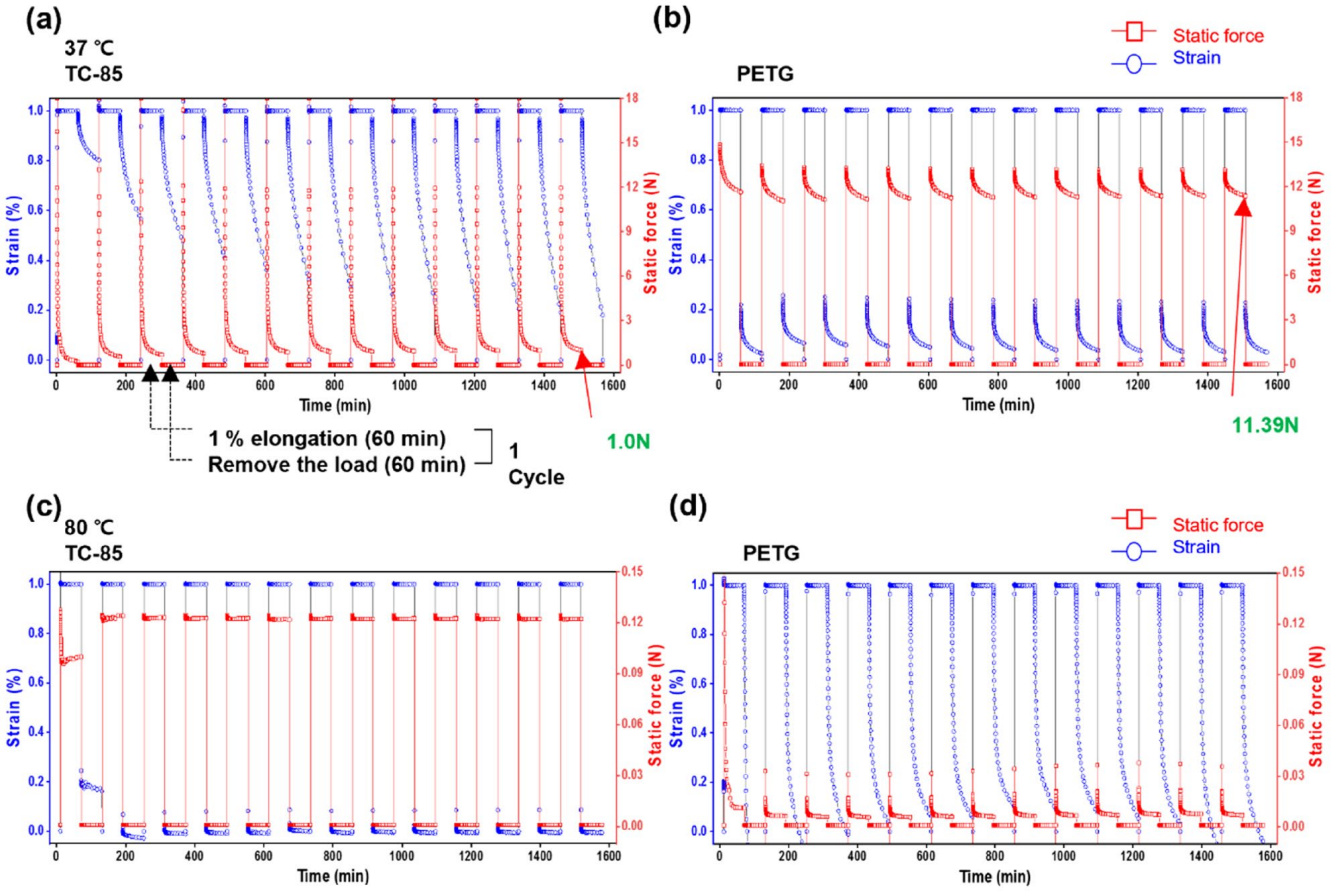
Stress relaxation and creep at 37 °C and 80 °C; The cycle of 1% elongation for 60 min and recovery for 60 min was repeated for13 cycles; (**a**) 37 °C, TC-85; (**b**) 37 °C, PETG; (**c**) 80 °C, TC-85; (**d**) 80 °C, PETG.

At 37 °C, TC-85 showed a static force of approximately 18 N at the start of elongation, followed by a rapid stress relaxation, which reduced the static force to 1.0 N. As the cycle was repeated, the amount of stress relaxation decreased, and the residual static force, the force that remained in the specimen after relaxation, increased. When the load was removed, the strain gradually recovered, and as the cycle was repeated, the rate of recovery and the amount of recovery gradually increased (Fig. 2a). PETG showed an initial static force of approximately 13 N, and the static force gradually decreased to 11.39 N. When the load was removed, 80% of the strain was instantaneously recovered, while the remaining 20% gradually recovered. Under cyclic loads, the residual static force and strain recovery rate of PETG also slightly increased; however, it was not increase as significantly as those of TC-85 (Fig. 2b).

#### Stress relaxation and creep at 80 °C

At 80 °C, TC-85 showed an initial static force of approximately 0.13 N, which was maintained with minimal force decay owing to stress relaxation. Even in repeated cycles, the residual static force after relaxation was constant. When the load was removed, most of the deformation was instantaneously recovered, and the recovery rate and recovery pattern were constant even with repeated load (Fig. 2c). PETG initially showed a static force of approximately 0.12 N, followed by a rapid stress relaxation, reducing the static force to 0.01 N. When the load was removed, the deformation was gradually recovered at 80 °C, which was unlike at 37 °C, where most of the deformation was instantly recovered. As the cycle was repeated, PETG showed a pattern where the remaining initial static force and residual static force after relaxation increased. Furthermore, the strain after recovered was negative (Fig. 2d).

### DMA

**Figure 3:**
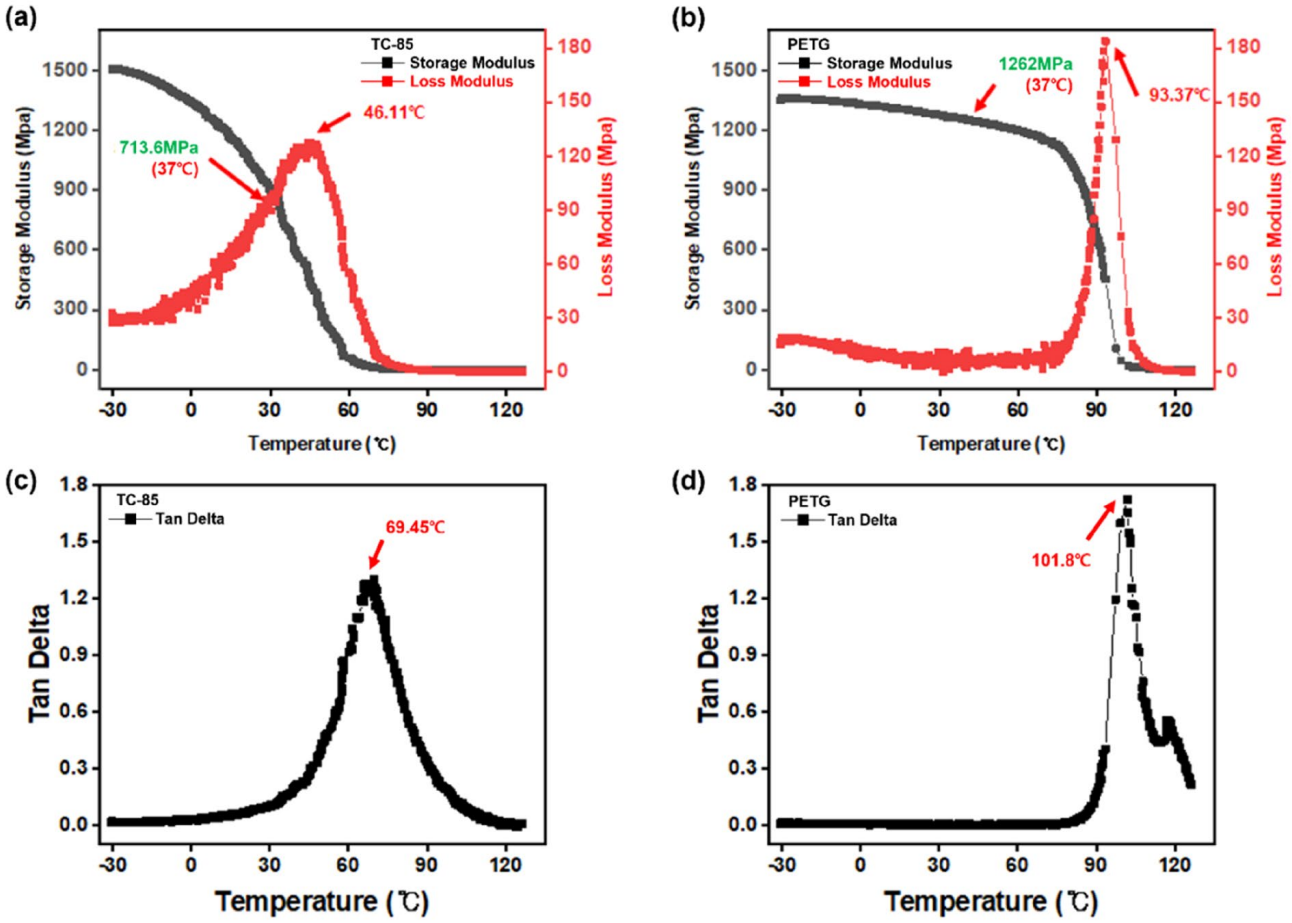
DMA temperature sweep; (**a**) storage and loss modulus of TC-85; (**b**) storage and loss modulus of PETG; (**c**) loss tangent of TC-85; (**d**) loss tangent of PETG.

Based on the DMA results, TC-85 showed a storage modulus, loss modulus, and loss tangent of 713.6 MPa, 111.60 MPa, and 0.16 at 37 °C, respectively (Fig. 3a,c). The storage and loss moduli of PETG were 1262 MPa and 5.58 MPa, respectively, with a loss tangent of 0.004 (Fig. 3b,d). Although both materials showed solid-like behaviour at 37 °C, TC-85 exhibited a more viscous response. In the present study, the T_g_ was defined as the peak of the loss tangent (tan δ), which is the ratio of the loss modulus to the storage modulus. The T_g_ of TC-85 and PETG were 69.45 °C and 101.8 °C, respectively (Fig. 3c,d).

### Shape memory property

**Figure 4:**
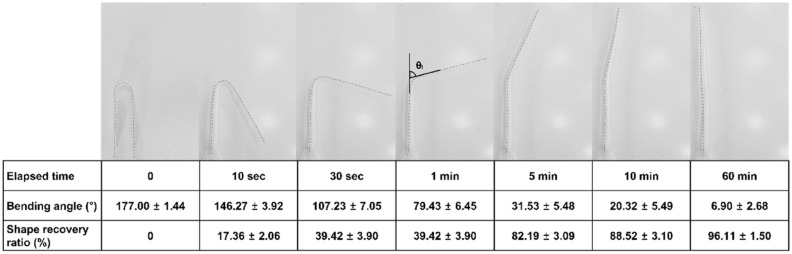
Shape memory effect and shape recovery ratio over time of TC-85 (N = 6).

After being bent at 80 °C, which is higher than its T_g_, the cooled TC-85 specimen remained folded (Fig. 4). However, the specimens recovered their original shape over time at 37 °C. A rapid recovery of more than 50% of the bending was observed within the first minute; however, the shape recovery rate gradually decreased thereafter. Approximately 90% of the deformation was recovered in 10 min, and the shape recovery ratio after 60 min was 96% (Fig. 4). Under the same conditions, PETG maintained its deformed shape and showed no shape recovery.

## Discussion

In the present study, the thermo-mechanical properties of the photocurable resin TC-85, a newly developed material for direct 3D printed clear aligners, were evaluated by comparing them with those of PETG, which is a widely used thermoplastic material for fabricating conventional clear aligners.

The thickness of the PETG specimens thermoformed on the standardized maxillary incisors model was 0.41 mm, which was only 54.7% of the original thickness before thermoforming (0.75 mm). This behaviour has been previously corroborated. Min et al. reported a 57.5% decrease in thickness of the materials that were thermoformed on a standardized maxillary incisors model representing the volume of teeth applied in clinical practice^13^. Further, a study of thermoforming thermoplastic materials using a square block with a thickness of 10 mm reported a thickness decrease by approximately 74.9–92.6%^12^. Therefore, it is apparent that conventional thermoplastic materials show various thickness changes after thermoforming, depending on the size and shape of the models.

In clinical practice, since clear aligners are manufactured using dental models of patients with different anatomical structures such as teeth size, dental arch size, alveolar bone height, and palatal vault depth, irregular thickness changes may occur. It has also been demonstrated that within a clear aligner, the thickness varied for each region such as the incisors, molars, and edentulous areas^22^. Since the thickness of the aligners is the factor that has the greatest influence on the orthodontic force applied to the teeth^13,23^, the irregular thickness of the clear aligners makes it difficult for clinicians to predict the performance of the clear aligners and the outcomes of the treatment. If the clear aligners are manufactured by 3D printing, it is possible to decrease the variation in thickness and increase the predictability of treatment outcomes. Moreover, if the technology is further developed, the thickness can be modified according to the type of tooth movement and regions, in turn potentially increasing the efficiency of the treatment in clinical practice.

Currently, 3D printed clear aligners using TC-85 material are generally manufactured by a digital lighting processing (DLP)-type 3D printer with layer thickness set to 100 μm. Residual resin remaining on the surface of the printed aligners is removed using a soft scraper and alcohol. Finally, they are post-cured under N_2_ with UV light using a post-curing chamber. The specimens of TC-85 in this study were manufactured by the same procedure (detailed procedures are described in the “Materials and methods”).

The thickness of the printed TC-85 specimens was 0.56 mm, which was 12% thicker than the set value (0.5 mm). Because the DLP-type 3D printer cures the liquid photosensitive resin using a high-definition projector as a light source, the resin can be polymerized thicker than the set value. A previous study described that the over-penetrance of light in 3D printing with transparent materials can induce overbuilding^24^. In addition, although the liquid resin on the surface of the specimens was mechanically removed after 3D printing, residual resin that remained was polymerized during the post-curing process^18^, which could result in a thicker specimen.

Overbuilding during 3D printing prevents the clear aligners from being fully seated on the teeth. In addition, excessive orthodontic force may be applied to the teeth by the clear aligners^18^. Overbuilding can be reduced and accuracy can be increased by setting the layer thickness of the 3D printer to 50 μm or printing with a stereo lithography apparatus (SLA)-type 3D printer^25^. However, because SLA-type 3D printers print each part with a laser source, it takes a longer time to fabricate the whole object. It is difficult to apply the SLA-type printer in clinical practice where multiple stages of clear aligners must be manufactured.

When 3D printing the clear aligners, the offset should be considered to improve the fit of the aligners. For example, in a study where splints were manufactured by the DLP-type 3D printer, a higher fit was observed when the offset was 0.1 mm^26^. Therefore, to apply the newly developed TC-85 to clinical practice, further studies are required to establish the offset setting, calibration, and workflow conditions.

The yield strength and elastic modulus were significantly higher in PETG than in TC-85 (Fig. 1), while the elastic range was significantly larger in TC-85 (4.65%) than in PETG (3.92%). With conventional clear aligners manufactured by thermoplastic materials, it is recommended to perform tooth movement of 0.25–0.33 mm per step and activate it once every two weeks for effective orthodontic tooth movement^21,27^. These results indicate that owing to the higher flexibility and larger elastic range of the clear aligners manufactured using TC-85, more tooth movement can be performed per step without permanent deformation.

Regarding the stress relaxation and creep behaviour, at 37 °C, when a load was applied for 60 min, TC-85 initially showed a rapid stress relaxation; after 13 cyclic loads, a residual static force of 1.0 N was observed. Although the stress relaxation occurred in PETG, the amount of relaxation was smaller than that of TC-85, with a residual static force of 11.39 N after 13 cyclic loads. The stress relaxation and creep behaviour of TC-85 offers these clear aligners with greater flexibility and improved fitting after being worn in the oral cavity^28^.

In addition, by constantly applying a light force to the teeth, it is possible to induce a physiological movement of the teeth^29^ and reduce the discomfort of the patient. In clinical practice, an orthodontic force of 0.098–1.18 N is recommended depending on the types of tooth movement^30^. Excessive force may have side effects on the teeth and surrounding tissues, including root resorption, and an orthodontic force applied beyond the pain threshold of the patients will result in discomfort^13^. The static force shown by TC-85 at 37 °C was appropriate to apply an orthodontic force. However, the large initial static force of TC-85 may induce discomfort to the patients when inserting the aligner. Also, since a large amount of stress relaxation occurs, the predictability of the aligner may deteriorate.

In TC-85, as the load cycle was repeated, the rate of strain recovery increased and the residual static force after relaxation also showed a gradual increasing pattern. The crystallization in the amorphous portion of the material by tensile stress may induce this behaviour^31–33^. In PETG, the residual static force and strain recovery rate remained relatively constant even after repeated cyclic loads. In clear aligners, the force decay is generated because of the viscoelastic properties of the material and the permanent deformation caused by repeated insertion of the aligners. It has been previously shown that a three-point bending test on materials of the clear aligners resulted in a 10–17% decrease in the static force after repeated loads^13^. The creep behaviour of TC-85, with the gradually increasing static force under cyclic loads, may be more advantageous in respect to clinical performance, as force decay is reduced, maintaining the orthodontic force of the aligners.

The creep and stress relaxation behaviours of TC-85 and PETG at 80 °C differed from those at 37 °C. TC-85 showed a slight stress relaxation, and after 13 cyclic loads, the static force was 0.12 N, whereas PETG exhibited a rapid stress relaxation and showed low static force as 0.01 N. At 80 °C, the interaction between the polymer chains of both materials is weakened; therefore, their storage modulus and elasticity decrease. However, owing to the cross-linked structure of TC-85, it is highly stable; therefore, it was able to maintain a constant stiffness while also retaining the static force and strain recovery patterns after repeated loads. PETG, a non-cross-linked polymer, showed a pattern of gradual increase in the static force under the cyclic loads and the strain recovered to a negative value. At 80 °C, the interactions between the polymer chains of PETG are weakened, allowing the movement of chains; therefore, thermal shrinkage was generated^32,34^.

TC-85 has geometric stability at high temperatures without heat shrinkage as demonstrated, which can be an advantage in clinical practice. Manufacturing clear aligners with TC-85 renders the hygiene management and disinfection of the aligner feasible. Generally, microorganisms begin to colonize the clear aligner surface 6 h after inserting the aligner^35^, and the deposited biofilm prevents the full coverage of the dentition with the clear aligners, thereby resulting in unaccomplished tooth movement^36^. However, clinicians advise patients not to clean or disinfect the aligners at high temperatures because the thermoplastic materials such as PETG deform. A thermophysiology study has indicated that *Streptococcus mutans* and related lactic acid bacteria, which are the major causative organisms for dental caries, are inactivated at temperatures above 60 °C^37^. Further studies are required to verify that washing the aligners at high temperatures does not affect their performance and has a disinfection effect in actual clinical practice.

As determine by DMA, the T_g_ of TC-85 (69.85 °C), estimated through the peak of the loss tangent (tan δ), was approximately 30 °C lower than that of PETG (101.8 °C). Although PETG is a non-cross-linked polymer, it contains aromatic rings; thus, the polymer chains form a strong pi-pi stacking interaction^38^. The polymer chains of TC-85 form a cross-linked structure through a difunctional oligomer, owing to the low molecular weight of the monomer, and the interaction between the polymer chains is weaker than that of PETG. Therefore, at 37 °C, TC-85 showed a lower storage modulus, a larger loss tangent (tan δ), and lower elasticity than PETG. Therefore, TC-85 displayed viscous behaviour, i.e., the strain gradually recovered upon removing the load and a large amount of stress relaxation was observed. At 80 °C, the interaction between the polymer chains decreases; therefore, the storage moduli of TC-85 and PETG reduced to 3.62 MPa and 1043.50 MPa, respectively. Although the storage modulus of PETG was significantly higher than that of TC-85, unlike the latter, PETG does not have a cross-linked structure that can restrain chain movement, resulting in a significant increase in the amount of stress relaxation. Furthermore, as movement between chains became possible, thermal shrinkage occurred. In contrast, TC-85 maintained a constant static force and strain recovery because the cross-linked structure stabilized the movement between the chains.

**Figure 5:**
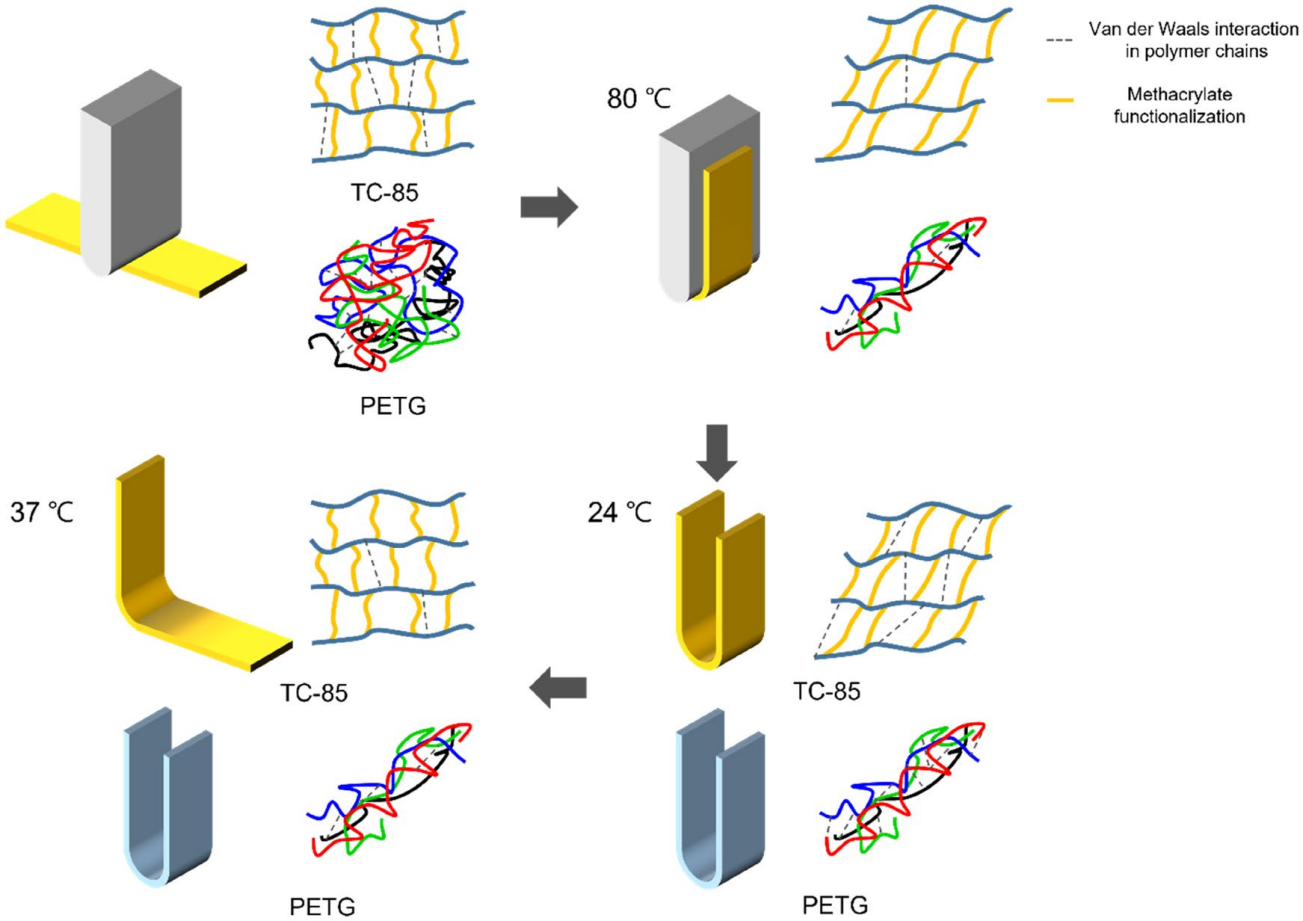
Shape memory property test procedure and shape memory mechanism; Yellow and blue specimens indicate TC-85 and PETG, respectively.

At a higher temperature of 80 °C, the Van der Waals interaction was significantly weakened and the polymer chain movement became possible. At a lower temperature of 24 °C, the Van der Waals interaction was re-formed and the shape of the specimens was fixed. Although 37 °C is lower than the T_g_ of TC-85, sufficient energy was supplied to enable the polymer chain movement; in addition, frozen stress was released. TC-85, which was cross-linked like a mesh, realized shape recovery^39^. Approximately 90% of the deformation rapidly recovered to its original shape within 10 min, and after 60 min the shape recovery ratio was 96%, exhibiting excellent shape memory properties. However, PETG, which is not cross-linked, like a thread, did not recover its original shape (Fig. 5).

Owing to the shape memory property of TC-85, the aligners can constantly apply orthodontic forces to the teeth under normal body temperature conditions without force decay caused by the deformation of the aligners. It also presents an advantage when the patients wear the aligners. Before applying the aligners, patients can immerse them in warm water to render them flexible, which can reduce the discomfort upon wearing them and provide a better fit. Even if the aligner is deformed along the dentition, its original printed shape and stiffness will be recovered at 37 °C. Therefore, the aligners can apply a constant orthodontic force to the teeth. In this study, the shape memory property was qualitatively evaluated and the property was visualized through a bending test. However, further studies such as the tension test are required in the future.

This study evaluated the basic mechanical properties of the photocurable resin TC-85, a newly developed material for 3D printed aligners, and only one type of the thermoplastic materials, PETG, was compared as a control. Therefore, it is difficult to conclude that TC-85 is superior to all the conventional materials used for manufacturing the clear aligners. However, since all conventional thermoplastic materials are non-cross-linked polymers, the variation in mechanical properties according to temperature will show a relatively similar pattern. They are not geometrically stable at high temperatures and have no shape memory property. Therefore, TC-85 presents favourable properties and demonstrates significant advantages for the clinical application of aligners.

## Materials and methods

### Specimen preparation

**Figure 6:**
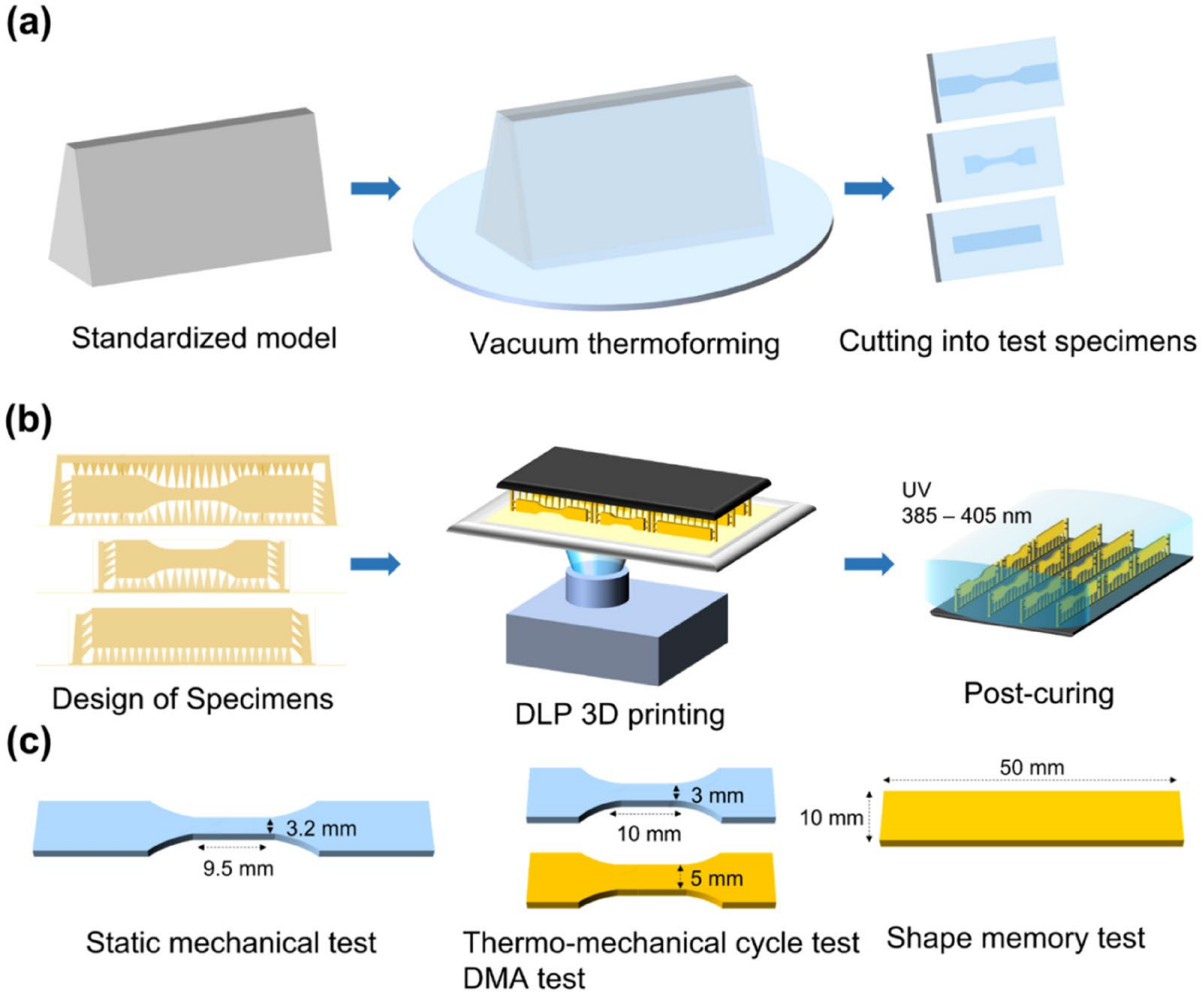
The preparing process and size of specimens; (**a**) Thermoplastic material (PETG); (**b**) 3D printed photocurable resin (TC-85); (**c**) size of specimens.

PETG (Easy-Vac gasket, 3A MEDES, Korea) with 0.75 mm thickness was used as the thermoplastic material for the clear aligners. In consideration of the clinical manufacturing environments, a standardized model of the maxillary central incisors in Korean adults was designed. The model was constructed by applying a tooth model (height = 20 mm) proposed by Sheridan et al.^40^, reflecting an average thickness of the incisal edge at 2 mm, a thickness of the height of contour at 8.5 mm, and a clinical crown height at 7 mm^13^. The designed standardized model was printed with a 3D printer (Uniz 4K, Uniz, USA) using S-100 (Graphy Inc., Korea) material. Then, PETG was vacuum thermoformed on the standardized model using a thermoforming caster (Ministar S, Scheu-Dental Gmbh, Germany) under the conditions recommended by the manufacturer. Specimens were prepared by cutting a flat surface of the thermoformed PETGs (Fig. 6a).

For the 3D printing, GR30860 and GR3060 (TC-85, Graphy Inc., Korea) were used as the oligomer resins, and bis(2,4,6-trimethylbenzoyl)-phenylphosphine oxide (Irgacure 819, BASF, Germany) was used as the photoinitiator. The specimens were designed according to each physical evaluation test standard, and the thickness was set to 0.5 mm considering that the thickness of PETG (0.75 mm) would decrease after thermoforming. The angle of the specimen was set at 90° to the horizontal plane to ensure that the support structure could only be on the side of the designed specimens. Specimens were printed with a DLP-type 3D printer (Uniz 4K, Uniz, USA) with the layer thickness set to 100 μm. Residual liquid resin remaining on the surface of the printed specimens was removed with a soft scraper, and then photocured twice for 25 min under N_2_ with UV light (wavelength: 385–405 nm) using a post-curing chamber (CureM U102H, Graphy Inc., Korea) (Fig. 6b).

Dumbbell shaped specimens (3.2 × 9.5 mm) were prepared for the static mechanical test according to ASTM D638-5. The size of the specimens for the thermo-mechanical cycle and DMA tests was determined considering that the driving motor of the DMA equipment (Q800, TA instrument, USA) has a maximum capacity of 18 N: 3 × 10 mm for PETG, and 5 × 10 mm for TC-85. Specimens for the shape memory test were prepared in the form of a rectangular strip with a size of 10 × 50 mm. The average thickness of the specimen was obtained by measuring the thickness at three points (in the middle and at both ends) using a digital calliper (GAU-178.00, Eurotool, Inc., USA) (Fig. 6c).

### Static mechanical property test (tensile test)

Six specimens per group were subjected to a static tensile test at 25 °C and 55% humidity using a universal testing machine (AllroundLine Z010, 2 kN load cell, Zwick, Germany). The crosshead speed was set at 5 mm/min, and the specimen was elongated at a constant speed until completely fractured. The static mechanical properties of each material were evaluated by comparing the yield strength and elastic modulus using the Mann—Whitney test with a statistical software (IBM SPSS Statistics for Windows, version 20.0, IBM Co., USA).

### Thermo-mechanical cycle property test (stress relaxation and creep test)

Repeated creep behaviour and stress relaxation of each material were evaluated by DMA under the stress relaxation mode. Since the mechanical behaviour of the materials changes dynamically around the transition temperature, two experimental temperatures were chosen through preliminary experiments: 37 °C, to mimic the temperature in the oral cavity; 80 °C, to mimic the temperature of hot food or beverage, which are frequently consumed in daily life. Generally, the aligners encounter a condition between the two set temperatures. At 37 °C and 80 °C, the cycle of 1% elongation for 60 min and recovery for 60 min was repeated 13 times. The stress relaxation and strain recovery patterns of the materials in response to time were evaluated. Three specimens were tested for each material, and it was estimated whether the results showed a deviation within 10%.

### Dynamic mechanical analysis test (temperature sweep)

To investigate the thermal dynamics of each material, DMA was performed between − 30 and 130 °C with a frequency of 1 Hz and strain rate 0.1% using the tensile method. The heating rate was set at 5 °C/min. Storage modulus, loss modulus, and loss tangent in response to temperature were measured. Three specimens were tested for each material, and it was estimated whether the results showed a deviation within 10%.

### Shape memory property test

Among several methods that can evaluate the shape recovery effect of materials, the bending test was chosen to qualitatively investigate and directly visualize the property^39,41^. A U-shaped model was prepared to standardize the case in which a tooth deviates from the line of occlusion to the buccal side and a bending force is applied to the aligners. The central shaft of the U-shaped model was 4 mm. The specimens were bent into a U-shape according to the model at 80 °C, which was higher than the transition temperature (T_g_) of TC-85, and maintained for 5 min (note: the T_g_s of both materials were determined by DMA). The specimens bent at the high temperature were rapidly cooled to 24 °C and maintained for 5 min. After removing the external force, the bending angle (θ_initial_) of the folded specimens was recorded. The specimens were immersed in a water bath at 37 °C and the shape recovery effect of the specimens was recorded with video at a frame rate of 30 FPS for 1 h. The initial bending angle (θ_initial_) and the bending angle (θ_t_) of the specimen at 10 s, 30 s, 1 min, 5 min, 10 min, and 60 min were measured using a mathematics program (GeoGebra, Markus Hohenwarter). The shape recovery ratio at 37 °C was determined using the following Eq. (1):

1 $$ {\text{Shape recovery ratio }} = \frac{\uptheta_{{\rm initial}} - \uptheta_{t}} {{\uptheta_{{\rm initial}} }} \times 100\%. $$

## Data Availability

The authors confirm that the data supporting the findings of this study are available within the article.
